# High Calorie, Low Nutrient Food/Beverage Intake and Video Gaming in Children as Potential Signals for Addictive Behavior

**DOI:** 10.3390/ijerph8124406

**Published:** 2011-11-29

**Authors:** Mary Ann Pentz, Donna Spruijt-Metz, Chih Ping Chou, Nathaniel R. Riggs

**Affiliations:** Department of Preventive Medicine, Institute for Prevention Research, University of Southern California, 2001 N. Soto Street, Los Angeles, CA 90033, USA; Email: dmetz@usc.edu (D.S.-M.); cchou@usc.edu (C.P.C.); nriggs@usc.edu (N.R.R.)

**Keywords:** eating, video gaming, children, addictive behavior

## Abstract

Little is known about the co-occurrence of health risk behaviors in childhood that may signal later addictive behavior. Using a survey, this study evaluated high calorie, low nutrient HCLN intake and video gaming behaviors in 964 fourth grade children over 18 months, with stress, sensation-seeking, inhibitory control, grades, perceived safety of environment, and demographic variables as predictors. SEM and growth curve analyses supported a co-occurrence model with some support for addiction specificity. Male gender, free/reduced lunch, low perceived safety and low inhibitory control independently predicted both gaming and HCLN intake. Ethnicity and low stress predicted HCLN. The findings raise questions about whether living in some impoverished neighborhoods may contribute to social isolation characterized by staying indoors, and HCLN intake and video gaming as compensatory behaviors. Future prevention programs could include skills training for inhibitory control, combined with changes in the built environment that increase safety, e.g., implementing Safe Routes to School Programs.

## 1. Introduction

As discussed in the first paper of this special issue of the *International Journal of Environmental Research and Public Health*, health risk behaviors which are potentially addictive follow a particular course, from initial appetite for the behavior, preoccupation with the behavior, loss of control over the behavior, negative consequences of continued behavioral practice, and lack of ability to stop the behavior [1]. DSM IV captures this progression in its diagnostic criteria for addictive behavior, and classifies actual addiction as extreme repetitive practice of a behavior to the extent that normal daily living functions are disrupted and the behavior produces harm to the individual [2]. DSM IV also considers co-morbidities associated with addiction. Whether separate addictions are co-occurring, or even co-predictive, is another question and one that is being addressed in the various papers in this issue. 

Historically, addictions have been examined as addictions to substance use, *i.e.*, tobacco, alcohol, and/or other drug use [3]. However, there is growing evidence that other health risk behaviors may also exhibit addiction “propensity.” In a review of 83 studies, Sussman *et al.* [4] concluded that eight behaviors in addition to substance use could be potentially addictive: binge eating, gambling, internet use, love, sex, exercise, work, and shopping. Video gaming has also gained recent attention as a potentially addictive behavior [5,6,7]. At least two of these potentially addictive behaviors, frequent or excessive high calorie, low nutrient (HCLN) food/beverage intake (which could relate to binge eating) and video gaming (a sedentary activity) have particular relevance to obesity, one of the foremost and escalating health problems in the world today [8,9,10,11].

### 1.1. Addictive High Calorie, Low Nutrient Food Intake and Video Gaming as Risk Factors for Obesity

The significant, global rise in obesity rates poses multiple and costly problems for health and society, including increased risk for cancer, heart disease, and diabetes, among other diseases [8]. Excessive HCLN intake, *i.e.*, high fat, high sugar snack foods and beverages, and video gaming (as part of sedentary behavior) have been shown to be significant risk factors for obesity [9,10,11,12]. Identifying the developmental course of HCLN intake and video gaming *before* these behaviors become addictive, and sufficiently early in life, could have important implications then for preventing both obesity and substance use. 

### 1.2. Obesity Risk in Childhood

Obesity risk escalates during the childhood years, commensurate with stages of development that are associated with adiposity rebound [13]. One of these stages is childhood [14]. HCLN intake has been shown to be a predictor of obesity at this age and later in life [14]. Sedentary behavior is another predictor of obesity in childhood [10,13]. Video gaming is considered one of several types of sedentary behavior that is usually evaluated together with television viewing, internet use, and inactivity [11,12,15]. There is relatively little research on the potential addictive progression of video gaming as a separate sedentary behavior in childhood. One of the few studies, conducted on students in grades 3,4,7 and 8, showed that increased frequency of gaming, low social competence, and high levels of impulsivity were associated with progression to pathological gaming over a two year period [5]. However, whether progression differed by grade level or age was not reported. 

No studies have been reported on addictive progression of HCLN intake in children, and none on the potential co-occurrence of video gaming and HCLN intake as addictive behaviors in this age group. Additionally, while there is some research on predictors of early sedentary behavior and HCLN intake frequency in children, including parental modeling, family rules, and feeding patterns, there is no research on predictors of *growth* in these behaviors in this age group that might signal addiction propensity (see [9,13]). Identifying predictors of addiction propensity for these behaviors in children, as well as addictive progression of these behaviors, could have significant implications for designing early addiction prevention programs. 

### 1.3. Predictors of Video Gaming and High Calorie, Low Nutrient Food Intake in Children

With virtually no studies available on predictors of propensity for addictive video gaming or HCLN intake in children, identifying potential predictors depends on findings from addiction studies on other age groups, most prominently, adolescents, and on other addictive behaviors that may share common risk factors with video gaming and high calorie, low nutrient food intake, most prominently, substance use. These include, but are not limited to: *early or frequent substance use relative to peers, high sensation-seeking, low impulse control, high stress, poor coping sills, poor school achievement, male gender, white race/ethnicity, and low socioeconomic status.*

An extensive body of research on adolescents has shown that early and frequent substance use, high sensation-seeking, and low impulse control consistently predict progression of substance use [16,17,18,19,20,21,22]. Increasingly, low impulse control has been interpreted as a deficit in one aspect of a larger set of cognitive-emotional functions referred to as executive cognitive function (ECF, [22,23,24]), which is linked with brain neurocircuitry [25,26,27]. Animal and human studies on adults have shown that physical obesity and substance use share common risk pathways through at least two areas of the brain: the pre-frontal cortex, particularly as relates to affective decision-making involving arousal and craving; and the nucleus acumens, particularly as relates to delay of reward or reinforcement of a behavior [28,29,30,31,32,33]. Inability to manage arousal and delay immediate reinforcement is observed as problems in ECF, more specifically as an inhibitory control deficit [34,35].

Other predictors have also shown relationships to adolescent substance use, particularly with progression from experimental to more regular, and potentially, more addictive use. Among these are high stress and low ability to cope with anxiety, and poor school achievement [28,36,37]. Demographic variables of gender, race/ethnicity, and socioeconomic status have also been shown to relate to substance use, although the direction of relationships varies somewhat by type of substance used [38,39].

Several of the risk factors found to predict adolescent substance use have also been found to predict obesity risk in children, if not specifically HCLN intake or video gaming. For example, obesity risk status, based on anthropometric measures of body mass index or waist circumference, is positively related to Hispanic and/or African-American race/ethnicity, gender (female, especially after puberty; male Hispanic), low socioeconomic status, inhibitory control deficit, poor grades, stress, and low coping ability [40,41,42,43,44]. Whether these variables also predict video gaming and high calorie, low nutrient food intake in children is not yet clear, although our previous study of latent classes of obesity risk indicated that in high obesity risk classes, male and Hispanic children were more likely than female and white children to engage in both HCLN and sedentary behavior (measured as total screen time) [40]. The Gentile *et al*. study [5] found that in the mixed sample of children and adolescents, progression to pathological gaming was associated with male gender, as well as later increases in anxiety/stress, depression, and social phobia—a potential indicator of social isolation—and decreased school achievement.

### 1.4. Objectives of the Present Study

In keeping with the intent of this special issue on co-occurring and co-morbid addictive behaviors, the present study tested four hypotheses pertaining to video gaming and high calorie, low nutrient food intake as potentially addictive behaviors in children. The first hypothesis was that video gaming and high calorie, low nutrient food intake would grow over time. The second hypothesis was that both video gaming and HCLN would co-occur within individuals. The third hypothesis was that both outcomes would have a common set of risk factors, based on variables derived from previous research on adolescent substance use and child obesity risk. The common risk factors were hypothesized to be inhibitory control problems, male gender, Hispanic race/ethnicity, low socioeconomic status, poor grades, high stress, and poor coping. The fourth hypothesis was that there would be some risk factors that differentiate the two behaviors, signaling potential addiction specificity. This hypothesis was exploratory, since there is relatively little research in children on whether specificity of risk factors would take the form of a difference in strength of risk or an opposite relationship of risk. 

## 2. Methods

### 2.1. Background

The present study used data from the baseline, six month follow-up, and 18-month follow-up waves of measurement in a randomized controlled trial for prevention of substance use and obesity in children, Pathways for Health, hereafter referred to as Pathways. The objective of Pathways is to translate two evidence-based programs for violence and substance use prevention, the Midwestern Prevention Project, or STAR [45], and PATHS [46] to a substance use and obesity prevention program for children, targeting schools that might represent higher risk for obesity by virtue of their Hispanic/Latino and low socioeconomic status representation. A total of 28 elementary schools in Southern California, including Title 12 schools (receiving federal aid) and schools with higher proportions of Hispanic/Latino students, from two Southern California school districts were matched in pairs on school-level demographic characteristics of achievement, size, ethnicity (% Hispanic/Latino), and socioeconomic status (% on free/reduced lunch) using the SAS RANUNI function [47] and then randomly assigned from within each pair to a school and parent-based program or control. Matching and randomization were conducted within each school district. 

### 2.2. Participants

At baseline, participants were 1,005 fourth grade (mean age 9.27 years) students from all 85 classrooms and 28 schools who had full active parent and self-consent for participation in the study. Of those 1,005, 96% (964) had complete data for study variables at baseline and constituted the current sample. Table 1 illustrates the sample characteristics for all study variables at baseline. Thirty-one percent were Caucasian, 27% were Hispanic/Latino and an additional, 8% Asian, 3% African American, 31% were either Hispanic multi-racial or “other.” Fifty percent were male and 25% reported receiving a free lunch at school. 

### 2.3. Measures

Participants completed a survey consisting of 145-items. The survey was administered aloud by a trained data collector, with a second data collector available to answer individual student questions about comprehension. Common to many school-based studies (e.g., [15]) data collection was constrained to one class period of approximately 45 minutes. Due to constraints of time, and possible constraints of comprehension and attention of fourth grade children, longer measures that had been previously developed and validated on adolescents were abbreviated in length, adapted for fourth grade reading comprehension, and re-validated. There is support in the psychometric literature for using abbreviated scales [48] and the practical reality of school-based prevention research is that assessment tools must be administered within the restrictions of time for school-based assessment. All procedures were approved by the University of Southern California Institutional Review Board. 

#### 2.3.1. High Calorie Low Nutrient Food/Beverage (HCLN) Intake

HCLN intake was assessed with five items taken from a validated open-source food frequency questionnaire [49]. The choice to select a subset of items from this questionnaire was based on constraints of survey length, as well as factor loadings on one factor representing HCLN. The five items included: How often do you drink soda—not diet (one can or glass); eat French fries or fried potatoes; eat corn chips, potato chips, popcorn, or crackers; eat doughnuts, pastries, cake, cookies (not low-fat); eat candy (chocolate, hard candy, candy bars) were selected by project investigators who have used these items in previous studies [41,42] and were compared to school teacher reports of food/beverage intake of their students as well as results of the California Healthy Kids Survey [50]. Abbreviated versions of food frequency questionnaires have demonstrated validity for fourth grade youth [48] and these specific items have been used with younger populations [41]. Response choices were 1 (Less than once a week), 2 (Once a week), 3 (2–3 times a week), 4 (4–6 times a week), 5 (Once a day), and 6 (2 or more of these a day). Internal consistency for the five items was adequate (α = 0.80).

#### 2.3.2. Video Gaming

Video gaming items were selected from the School-Based Nutrition Monitoring Student Questionnaire (NMSQ; 12). The two items were “On a regular school day, how many hours per day do you usually spend playing video games that you sit down to play like PlayStation, Xbox, GameBoy, or arcade games?” as well as “video games that make you move or breathe hard like Nintendo Wii?” Response choices ranged from 1 (“I don’t play videogames”) to 7 (“6 or more hours per day”). The mean of the two items was computed and the internal consistency for the two items was adequate (α = 0.74).

#### 2.3.3. Inhibitory Control Problems

Items from the Inhibit clinical sub-scale of the Behavioral Rating Inventory of Executive Function, Self-Report [34] were included to assess inhibitory control (e.g., “I do things without thinking first”). Item response choices ranged from 1 = Never, 2 = Sometimes, 3 = Often. Previous pilot studies, including a study of 107 fourth grade students, have demonstrated acceptable internal consistency for the full Inhibit scale (α = 0.78) [24,42]. For the current study, an abbreviated scale was constructed using the six highest loading index items from our pilot data. The abbreviated scale demonstrated predictive validity when compared to the full BRIEF-SR scale (α = 0.74). 

#### 2.3.4. Stress and Coping

Eight index items were selected from the Perceived Stress Scale (PSS) [51] based on our pilot analyses. The PSS contains items that tap reactivity to stressors (e.g., in the past week “I felt nervous or stressed”), as well as aspects of an individual’s capacity to cope with stressors (e.g., “I handled problems that bothered me”). A principal components analysis with promax rotation was conducted resulting in a two-factor (stress and poor coping) solution. The largest loading items were then selected to represent these factors. Internal consistencies were 0.59 and 0.67 for stress and low coping, respectively.

#### 2.3.5. Sensation Seeking

Three items were adapted from the Brief Sensation Seeking Scale for children and adolescents (BSS4 and SS2; e.g., “I like to do things that are a little scary;” 50). Based on a pilot study of 107 fourth grade children [24], language and scaling were simplified for comprehension (e.g., “frightening” was replaced with “scary;” a five point response choice was replaced with a three point response choice; from not at all = 1, very often = 5 to never = 1, often = 3). Three items were dropped due to low loadings in factor analyses, leaving a three-item scale. Internal consistency was α = 0.42, comparable to reports on the BSS4 (α = 0.44; [50,52]). 

#### 2.3.6. Perceived Neighborhood Safety

Children’s perceptions of neighborhood safety was assessed utilizing a single item from the Youth Risk Behavior Survey (YRBS) asking children “In the last month, have you ever not gone to school because you felt you would be unsafe at school or on your way to or from school?” [53,54]. Item response choices were reversed to represent 0 = no, 1 = yes.

#### 2.3.7. Covariates

Gender, ethnicity (African-American or Hispanic/Latino *vs*. Other), self-reported school grades, and socio-economic status (free lunch as proxy) were included as potential risk factors based on previous studies have shown some differences in sedentary activities and obesity risk by gender, ethnicity, and socioeconomic status [44,55].

### 2.4. Analysis Plan

All analyses were conducted using the individual as the unit of analysis and proceeded through a two-step process. Means and standard errors were computed in step one to describe the sample. In step two, growth curve analyses modeled relationships between independent variables and intercept and growth in HCLN intake and video gaming over an 18 month period (two school years, three waves of data). 

#### Growth Curve Analysis

Group differences in two parameters, the intercept and slope, were estimated for a growth curve model (GCM). The intercept represents the starting status of an individual’s use trajectory, from the first wave of data collection, in longitudinal observation. The linear slope represents the unidirectional trend of the change in high calorie snack food and video gaming across time. A GCM can be expressed as:

                                                        y_ij_ = a_j_ +t_ij_b_j_ +e_ij_

where y_ij_ represents the outcome measure for individual j at time i; t_ij_ is the time of measurement (e.g., t_ij_ = i – 1); while a_j_ and b_j_ stand for intercept and slope, respectively, and e_ij_ is normally distributed with mean 0 and variance σ^2^e. It is a_j_ and b_j_ that characterize the growth profile of an individual. 

Intercept and growth profiles were simultaneously estimated for HCLN intake and video gaming using Mplus 6.1 software with full information maximum likelihood imputation [56]. Mplus has the capacity to conduct analysis of complex survey data and yields standard errors and a chi-square test of model fit. Model fit indices for exploratory hypothesis testing included Chi-Square, Root Mean Square Error of Approximation (RMSEA), and Comparative Fit Index (CFI). Relationships among the two behaviors were modeled within each wave to evaluate a co-occurrence model, as well as across behaviors to evaluate a co-prediction model.

## 3. Results

### 3.1. Descriptive Characteristics

Demographic and behavioral characteristics of the sample at fourth grade baseline are shown in Table 1, expressed as means (for scaled variables), and percentages (for categorical variables). Mean values were used for growth curve analyses. Percentages are shown for descriptive purposes only, representing a relatively high level of risk or problem behavior where relevant (e.g., children who reported that they were “always” stressed for each of the three stress variables). The mean score for low inhibitory control indicates that on average, children report inhibitory control problems either “never” or “sometime,” with about 5% indicating “always” having inhibitory control problems for each of the 6 inhibitory items. The average for academic grades was between A’s and B’s, with 2.28% reporting that they received D’s or lower, representing poor school performance. On average children reported to be stressed between “never” to “sometimes” with almost 2% stating that they were “always” stressed for each of the three items. Children reported, on average, to “sometimes” be able to cope with stress as well as enjoy seeking sensation, with about 2% stating that they were “never” able to cope with stress and 2% stating that they “always” participated in sensation seeking activities. Almost 9% of children perceived their neighborhood to be unsafe enough so as to not go to school at least once in the last month. Children reported playing video games approximately 2.5 hours per day. Almost two-thirds of youth reported playing video games more than 20.5 hours per week. The cut-off of 20.5 hours per week for video gaming was based on Gentile *et al*.’s [5] analysis of ≥20.5 hours/week as representing a high level of video gaming, which far exceeds the 2 hours or more of daily television viewing that is typically considered as high risk sedentary behavior [15]. On average, children reported consuming each of five HCLN items between “once a week” and “2–3 times per week.” The cut-off used to illustrate high HCLN intake, ≥25 times per week, was arbitrary, based on a sum of frequency of daily consumption of different types of foods and beverages that would represent HCLN intake more than three times per day (≥25 times/week). 

**Table 1:** Behavioral and demographic characteristics of sample.

Variable	X (SE)	% (SE)
Inhibitory Control Problems	1.29 (0.01)	
Low Inhibitory Control		5.08 (0.01)
Grades	1.73 (0.02)	
Low Achievement		2.28 (0.00)
Stress	1.75 (0.02)	
High Stress		1.66 (0.00)
Coping	2.15 (0.02)	
Low Coping		1.76 (0.00)
Sensation Seeking	1.92 (0.01)	
High Sensation Seeking		2.28 (0.00)
White		30.50 (0.01)
Hispanic		26.97 (0.01)
African American		2.90 (0.01)
Asian		8.20 (0.01)
Mixed/Bi-Racial/Other		31.43 (0.01)
Free Lunch		23.34 (0.01)
Unsafe		8.60 (0.01)
Male		49.59 (0.02)
Video Gaming hours/day	2.45 (0.05)	
≥20.5 hours/week		62.96 (0.01)
HCLN ^†^ Intake	2.37 (0.03)	
≥25 Times Per Week		8.51 (0.01)

^†^ HCLN = High Calorie, Low Nutrient food/beverage intake. N = 964 fourth grade students with complete data.

Current nutritional guidelines, which could have been used to establish cut-offs, are based on % of caloric intake per day, which was not measured in this study (*cf.* [57,58]; http://www.cnpp.usda.gov/DGAs2010-PolicyDocument.htm). A total of 8.51% of the sample reported HCLN intake ≥25 times/week.

Table 2 presents correlations among study variables. As illustrated, inhibitory control problems and sensation seeking were positively correlated with video gaming and HCLN intake. Grades were negatively correlated with video gaming and HCLN intake. Stress was positively correlated with HCLN intake, and coping was not correlated with either video gaming or HCLN intake.

**Table 2:** Bivariate correlations among study variables.

	1	2	3	4	5	6	7	8	9	10
1. Inhibitory Control Problems										
2. Grades	-0.15***									
3. Stress	0.33**	-0.07*								
4. Coping	-0.13***	0.09**	-0.08*							
5. Sensation Seeking	0.33***	0.00	0.13***							
6. Hisp/AA	-0.04	-0.18***	-0.03	-0.02	-0.07*					
7. Free Lunch	0.00	-0.16***	0.01	-0.03	-0.02	0.27**				
8. Unsafe	0.11***	-0.12***	0.13***	-0.01	0.11***	0.07*	0.05			
9. Male	0.11***	-0.11***	-0.05	0.03	0.15***	0.05	0.01	0.02		
10. Video Gaming	0.15***	0.13***	0.04	-0.03	0.15***	0.06*	0.06	0.14***	0.32***	
11. HCLN Intake	0.13***	0.14***	0.11***	-0.05	0.11***	0.14***	0.18***	0.11***	0.17***	0.38***

*** = p < 0.001, ** = p < 0.01, * = p < 0.05. HCLN = High Calorie Low Nutrient Food/Beverage Consumption.

### 3.2. Estimates of Relationships of Predictors (Including Demographic Covariates) to HCLN Intake and Video Gaming

Fit estimates for the growth curve model were adequate (X^2^(325) = 685.68, p < 0.001; CFI = 0.950; TLI = 0.950; RMSEA = 0.034). Additionally, the variances for each of our intercept and growth parameters was significant (intercept and slope of video gaming, *p* < 0.01; intercept of HCLN, *p* < 0.01; slope of HCLN, *p* < 0.05). The independent relationships of each predictor to the intercept and slope of each outcome are shown in Table 3. Each outcome was tested in a separate model to determine whether the same set of predictors should be entered in subsequent growth curve analyses with both outcomes modeled simultaneously. As is shown in Table 3, the pattern of predictor/outcome relationships was similar for each outcome. Stress, grades, and coping showed relatively weak relationships to each outcome, but were marginally significant. All predictors were retained for subsequent analyses.

**Table 3:** Predictors of growth in HCLN intake and video gaming.

	HCLN Intake		Video Gaming	
Predictors	Intercept	Slope	Intercept	Slope
	*β (S.E.)*	*β (S.E.)*	*β (S.E.)*	*β (S.E.)*
Low Grades	-0.08 (0.04)†	0.00 (0.07)	-0.06 (0.04)†	-0.10 (0.05)
Male	0.18 (0.04)***	-0.26 (0.05)***	0.42 (0.03)***	0.03 (0.08)
Hispanic/AA	0.11 (0.04)**	0.16 (0.05)*	0.04 (0.04)	0.17 (0.09)†
Free Lunch	0.16 (0.04)***	0.07 (0.06)	0.08 (0.04)*	0.06 (0.09)
Unsafe Environment	0.09 (0.04)*	0.06 (0.07)	0.14 (0.04)***	0.02 (0.09)
High Stress	0.08 (0.04)†	-0.18 (0.05)*	-0.01 (0.04)	0.16 (0.09)†
Low Coping Skills	0.05 (0.04)	-0.06 (0.05)	0.07 (0.04)†	-0.10 (0.08)
Inhibitory Problems	0.11 (0.07)**	-0.08 (0.08)	0.13 (0.04)**	-0.28 (0.10)**

† = p < 0.10, * = *p* < 0.05, ** = *p* < 0.01; N = 964; HCNL = High Calorie, Low (Poor) Nutrient food and beverage intake.

### 3.3. Co-Occurrence and Growth in HCLN Intake and Video Gaming

**Figure 1:**
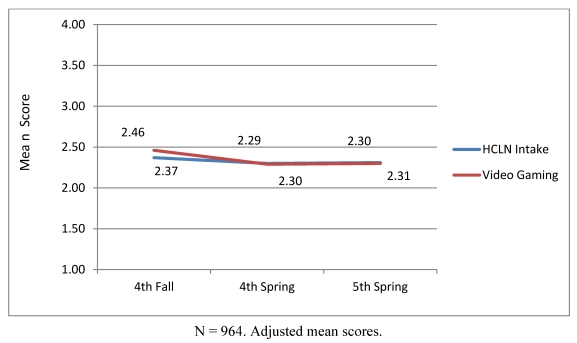
Co-occurrence and growth in HCLN and video gaming.

**Figure 2:**
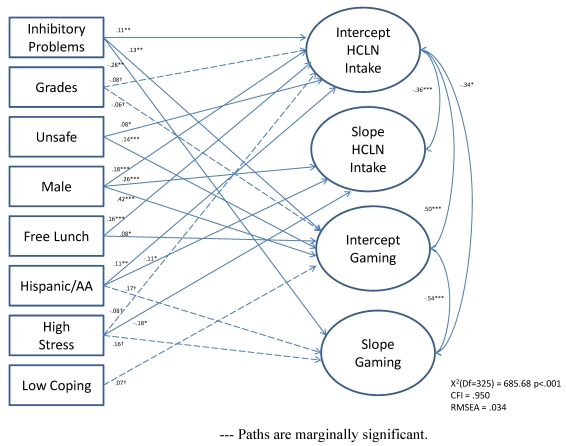
Growth curve model of predictors of HCLN intake and video gaming.

Figure 1 illustrates means for the dependent variables at each study wave, with similar patterns of growth for both HCLN and gaming. Figure 1 illustrates that study Hypothesis 1 was not supported: there was no significant growth in either HCLN intake or video gaming across study waves. Although we did not find growth in mean levels of video gaming and HCLN intake over the three waves of data, we did, as stated above, find significant variance in each of our intercept and, perhaps more importantly, growth parameters. Thus, there is the potential our independent variables to predict this variance. Figure 2 illustrates significant positive associations between video gaming and HCLN intercepts which supports Hypothesis 2 that the two health behaviors would co-occur. 

### 3.4. Common and Behavior Specific Predictors

As illustrated by Figure 2, inhibitory control problems, perceived neighborhood danger or lack of safety, being male, and free/reduced lunch status were each significantly and positively associated with both HCLN intake and video gaming intercepts. Being male was also positively associated with HCLN slope. Being either Hispanic or African-American was significantly associated with HCLN intake intercept and slope. High stress was negatively associated with HCLN intake slope. Inhibitory control problems were significantly negatively associated with video gaming slope. Neither school grades nor poor coping skills were significantly associated with growth parameters. Therefore, Hypotheses 3 and 4 were supported in that there were both common and specific predictors to substance use and HCLN.

## 4. Discussion and Conclusions

### 4.1. Summary

The present study tested four hypotheses, three of which were supported. The first hypothesis was that video gaming and HCLN intake grow over time in children. Rapid or extreme growth was assumed to represent potential for addictive behavior. Results of this study showed that high levels of video gaming and HCLN intake were apparent as early as fourth grade, a period of child development associated with adiposity rebound and increased risk for obesity, and that the two behaviors were highly correlated. However, from the starting point of high levels, there was little further growth in video gaming or HHhHCLN intake from fourth through fifth grade, although there was significant variance in the growth parameters for each behavior. There are several plausible explanations for lack of growth. One is that initially high levels may have produced a ceiling effect. A second is that the study examined growth over a relatively short period of time representing two grades and an 18 month period. A third is that growth was examined only during the elementary school years, representing a developmental period over which children may have relatively little individual choice in behavior. Future research could examine whether growth in both behaviors increases significantly once children move into middle school and the early adolescent years that are associated with increased parental autonomy, peer pressure, and individual choice. If so, the pattern would support the use of a piece-wise growth curve model of analysis that can estimate the effects of change in school environment as well as change in developmental stage (from childhood to adolescence).

The second hypothesis was that video gaming and HCLN intake would co-occur. Results of this study supported a co-occurrence model of video gaming and HCLN intake. Both the intercepts and the slopes of the behaviors were highly correlated. In addition, a higher level (intercept) of HCLN intake at baseline was associated with lower growth in video gaming over time (slope). This finding would appear to be counterintuitive but three possibilities could explain this finding. One possibility is that snacking might make video gaming difficult if both hands are occupied in operating video game controls. A second possibility is that since the growth in both behaviors was almost negligent, the finding may be an artifact of a ceiling effect produced by the high intercept values, as noted above. A third possibility is that high levels of HCLN intake may be occurring during periods of alternative sedentary activities such as television viewing or computer homework activities, or around school hours, during which video gaming would not be likely. The present study did not evaluate this possibility, although the correlations of video gaming with other sedentary activities were relatively high (TV watching hours as screen time, *r* = 0.50, *p* < 0.001; computer hours, *r* = 0.46, *p* < 0.001), and are consistent with findings from other research that has shown a negative correlational relationship between video gaming and length of time spent on exercise [11,12].

The third hypothesis was that there was a common set of predictors of both video gaming and HCLN intake. Based on previous research, these were inhibitory control problems, male gender, low socioeconomic status, poor grades, Hispanic race/ethnicity, high stress, and poor coping [5,38,39,40,41,42]. Four common risk factors were found, thus supporting the third hypothesis. The strongest predictors of both behaviors were inhibitory control problems, male gender, low socioeconomic status as measured by receiving free or reduced lunch at school, and an exploratory factor, perceived lack of safety of the neighborhood environment, which has received relatively little attention in the literature on addictive behaviors in children. Overall, the results are consistent with findings from adolescent substance use studies that have shown associations between inhibitory control problems, male gender, low socioeconomic status and substance use (e.g., [17,18]), as well as a previous study on children [24]. Inhibitory control deficit (similar to impulsivity) was significantly related to high intercept levels of both video gaming *and* HCLN intake. Boys exhibited higher levels of both video gaming and HCLN intake than girls, and slightly more growth in video gaming. Receiving free or reduced lunch, was positively related to intercepts and growth in both video gaming and HCLN intake. Additionally, perceived lack of safety in the environment representing from home to school was significantly related to high levels of both video gaming and HCLN intake. This may be the first study to relate safety to these co-occurring behaviors. Previous research on adults has focused on the relationships of lack of perceived safety of the neighborhood environment to low levels of walking as a physical activity [59]. 

The results of the present study raise the possibility that perceived lack of safety may keep children indoors at home, whether this is a personal decision or due to parent rules and concerns about safety. With few opportunities to engage in physical activity within the home, combined with potential boredom over being restricted in activity, children may turn to greater HCLN intake and video gaming as means to cope with confinement. In conjunction with the findings on socioeconomic status, results of this study raise the question of whether children who live in some types of impoverished, unsafe neighborhoods might constitute a maj or risk group for developing addictive gaming and HCLN intake behaviors, and subsequently, health problems related to these behaviors, including obesity and Type II diabetes [60,61]. 

The fourth exploratory hypothesis was that some risk factors differentiated video gaming and HCLN intake either in terms of strength or directionality which could signal potential addiction specificity. There were three. Hispanic or African-American status was positively related to intercept and growth in HCLN intake, but not related to gaming. High stress was negatively related to HCLN intake, but showed a non-significant positive relationship to growth in gaming. The direction of relationship of stress to HCLN intake is counter to findings on effects of stress and poor coping on binge eating in adults, as well as stressful, emotional eating as reported by adolescents [32,33,41]. One possible explanation for the contrary finding is that children may consume HCLN products because they may be readily available in the home rather than as a response to stress. The low prevalence of children in this study who reported high levels of stress would support this explanation. Finally, although the relationship was not significant, low coping was positively related to the gaming intercept, but not related to HCLN intake. 

### 4.2. Unexpected Findings

School achievement was not significantly related to either video gaming or HCLN intake, although there was a non-significant trend of lower grades associated with both higher video gaming and HCLN intake intercepts (*cf.* [5,38]). The Gentile *et al*. [5] study found a significant relationship of poor achievement to video gaming, however, achievement was examined as an outcome rather than as a predictor and the focus was pathological gaming rather than growth in gaming behavior. 

Another unexpected finding was the lack of relationship of sensation-seeking to either video gaming or HCLN intake in growth curve analyses. Although the initial correlations of sensation-seeking with these behaviors were significant, they were small (r = 0.16 with gaming, r = 0.11 with high calorie, low nutrient food intake, p’s < 0.05), and sensation-seeking was subsequently eliminated from further analyses because it did not contribute to model fit. It also showed poor internal consistency (α = 0.42), although comparable to that found for the BSS4 (α = 0.44; 50). One possible explanation is that much of previous research that has measured sensation-seeking and shown relationships of sensation-seeking to substance use and other health risk behaviors is based on adolescent populations (e.g., [17,18,19]). Arousal and impulsivity, which are associated with increased risk-taking and sensation seeking, appear to be linked with changes in brain circuitry during adolescence [62]. Furthermore, research suggests an increased neurobiological vulnerability to addictive behavior during adolescence [25,36]. These neurobiological changes may not have occurred yet in children. Thus, even if a child exhibited a high level of sensation-seeking, it may not yet operate as a neurobiological trigger to addictive behavior.

### 4.3. Limitations

There are several study limitations which should be considered in drawing conclusions about video gaming and HCLN intake as potentially addictive behaviors in childhood. One is reliance on self-report measures, several of which were abbreviated for use with children. However, the study used measures that have been standardized in other studies and abbreviated to accommodate to school class time restrictions, with comparable reliability [41,48,49,52]. Another is that the study period, although longitudinal with three waves of measurement, may not yet be sufficient to find significant growth in behavior. However, the focus on children for purposes of early prediction, combined with the finding of relatively high intercepts at baseline in fourth grade, should have important implications for both identifying and preventing addictive behavior propensity. An additional limitation is that other potential risk factors for video gaming and HCLN intake were not included in this study, primarily because there were no corresponding measures for both behaviors. Primary among these are parent influences [9]. While modeling of HCLN intake by parents is included in the Pathways trial, there are no corresponding variables available for video gaming. Thus these risk factors could not be evaluated in a co-occurrence or co-prediction model. 

### 4.4. Implications of the Findings and Future Directions

Several findings have particular importance for designing programs to prevent addictive behavior as early as in childhood. One is that video gaming and HCLN intake appear to co-occur in children and exhibit several common risk factors which are also associated with substance use behavior. This finding argues strongly for the development of universal prevention programs that are aimed at preventing multiple health risk behaviors early in childhood [9]. Second is the strong predictive relationship of low inhibitory control and low perceived safety to both HCLN intake and gaming. These findings suggest that a multiple health risk behavior prevention program should probably take a multi-level, ecological approach that incorporates individual skills training to improve executive cognitive function (ECF) [35], as well as physical exercise to replace sedentary activity or promotion of active rather than passive video gaming in the absence of other physical activity opportunities [11,12], and strategies to improve the safety of the built environment surrounding the child in order to facilitate walking and other types of outdoor exercise, for example, introduction of a Safe Routes to Schools program. There is already growing evidence to suggest that ECF training has multiple benefits for children [63], that increased physical activity has a positive effect on ECF [64] and negative effect on substance use [65], and that increasing perceived safety of the environment promotes more walking [59]. Whether increased walking can replace sedentary screen time, whether this involves gaming, television viewing, or internet or mobile use, is not yet known [66,67]. Finally, some factors that have been found to predict substance use in adolescents did not predict HCLN intake or gaming in children (low grades, sensation seeking, low coping), and others (race/ethnicity and high stress) had a differential effect on HCLN but not gaming. The variation in risk factors suggests that future programs that do include multiple health risk behaviors might tailor applications of skills training to different groups and different situational contexts. For example, addressing prosocial alternatives to sensation-seeking might be applied to substance use risk situations but not food choice situations; and addressing healthy food choices might be tailored to the context of different parent modeling behaviors or different food products that are available in some homes but not others. 
